# Pathogenic diversity amongst serotype C VGIII and VGIV *Cryptococcus gattii* isolates

**DOI:** 10.1038/srep11717

**Published:** 2015-07-08

**Authors:** Jéssica Rodrigues, Fernanda L. Fonseca, Rafael O. Schneider, Rodrigo M. da C. Godinho, Carolina Firacative, Krystyna Maszewska, Wieland Meyer, Augusto Schrank, Charley Staats, Livia Kmetzsch, Marilene H. Vainstein, Marcio L. Rodrigues

**Affiliations:** 1 Instituto de Microbiologia Paulo de Góes, Federal University of Rio de Janeiro, Rio de Janeiro, Brazil; 2 Fundação Oswaldo Cruz – Fiocruz, Centro de Desenvolvimento Tecnológico em Saúde (CDTS), Rio de Janeiro, Brazil; 3 Centro de Biotecnologia, Federal University of Rio Grande do Sul, Porto Alegre, Brazil; 4 Molecular Mycology Research Laboratory, Centre for Infectious Diseases and Microbiology, Sydney Medical School – Westmead Hospital, Marie Bashir Institute for Infectious Diseases and Biosecurity, The University of Sydney, Westmead Millennium Institute, Sydney, Australia; 5 Grupo de Microbiología, Instituto Nacional de Salud, Bogotá, Colombia

## Abstract

*Cryptococcus gattii* is one of the causative agents of human cryptococcosis. Highly virulent strains of serotype B *C. gattii* have been studied in detail, but little information is available on the pathogenic properties of serotype C isolates. In this study, we analyzed pathogenic determinants in three serotype C *C. gattii* isolates (106.97, ATCC 24066 and WM 779). Isolate ATCC 24066 (molecular type VGIII) differed from isolates WM 779 and 106.97 (both VGIV) in capsule dimensions, expression of *CAP* genes, chitooligomer distribution, and induction of host chitinase activity. Isolate WM 779 was more efficient than the others in producing pigments and all three isolates had distinct patterns of reactivity with antibodies to glucuronoxylomannan. This great phenotypic diversity reflected in differential pathogenicity. VGIV isolates WM 779 and 106.97 were similar in their ability to cause lethality and produced higher pulmonary fungal burden in a murine model of cryptococcosis, while isolate ATCC 24066 (VGIII) was unable to reach the brain and caused reduced lethality in intranasally infected mice. These results demonstrate a high diversity in the pathogenic potential of isolates of *C. gattii* belonging to the molecular types VGIII and VGIV.

Cryptococcosis is a fungal disease that kills approximately 650,000 individuals every year^1^. *Cryptococcus neoformans* and *C. gattii,* the etiological agents of this mycosis^2^, form the so-called *C. neoformans* species complex^3^. *C. neoformans* is cosmopolitan and commonly causes disease in immunocompromised individuals^4^, while *C. gattii* preferentially affects immunocompetent individuals particularly in tropical and sub-tropical regions^5^. *C. gattii* outbreaks have also been reported in the Pacific Northwest Region of the United States and in the Vancouver island^4^,^6^,^7^,^8^,^9^.

*C. gattii* is classified into four different molecular types (VGI, VGII, VGIII and VGIV)^10^,^11^ and two serotypes (B and C)^12^. Due to its detection in the US and Canada outbreaks, VGII is the most well characterized molecular type of *C. gattii*^13^,^14^,^15^. It has been described that virulent *C. gattii* VGII outbreak lineages derived from mating events in the Brazilian Amazon rainforest and then dispersed to temperate regions^16^,^17^,^18^. VGI isolates can also cause disease in healthy individuals^13^, while the VGIII molecular type has been associated with clinical syndromes in immunocompromised patients^19^. Isolation of VGIV isolates is, in general, less frequent, although this molecular type is considered to be endemic in the sub-Saharan Africa^11^. Importantly, it has been recently demonstrated that virulence is not specifically associated with a particular major molecular type of *C. gattii*, but rather with still unknown individual strain attributes^20^.

*C. gattii* and *C. neoformans* produce capsules mainly composed of polysaccharides, which are considered to be essential for cryptococcal pathogenesis^21^. The most abundant component of the capsule is glucuronoxylomannan (GXM), followed by glucuronoxylomannogalactan (GXMGal) and mannoproteins^22^. Minor, transitory capsular components have also been identified, including heat-shock proteins^23^, glucans^24^ and chitooligomers^25^. Both major and minor capsular components are believed to directly influence the interaction of cryptococci with the host. Recently, chitooligomers have been connected with the ability of *C. neoformans* to colonize the brain^26^.

The clinical relevance of VGII isolates of *C. gattii* has stimulated a number of studies focusing on how the fungus interacts with host cells^27^,^28^. On the other hand, the pathogenic properties of VGIII and VGIV isolates are known to a lesser extent. In this study, we have phenotypically characterized three serotype C isolates of *C. gattii* of the molecular types VGIII and VGIV. Our results suggest an important phenotypic diversity among these three *C. gattii* isolates, including differences in pigmentation, capsular serology, gene expression in response to capsule-inducing conditions, chitooligomer production *in vivo* and induction of chitinase activity in mice. These differences were correlated with the ability of *C. gattii* to kill infected mice.

## Results

### Phenotypic analysis

Although a revision of the *C. neoformans*/*C. gattii* species complex has recently been proposed^29^, we adopted in this manuscript the standard *C. gattii* classification into four molecular types, as the acceptance of the new taxonomic proposal in the *Cryptococcus* community is still uncertain^3^.

**Figure 1:**
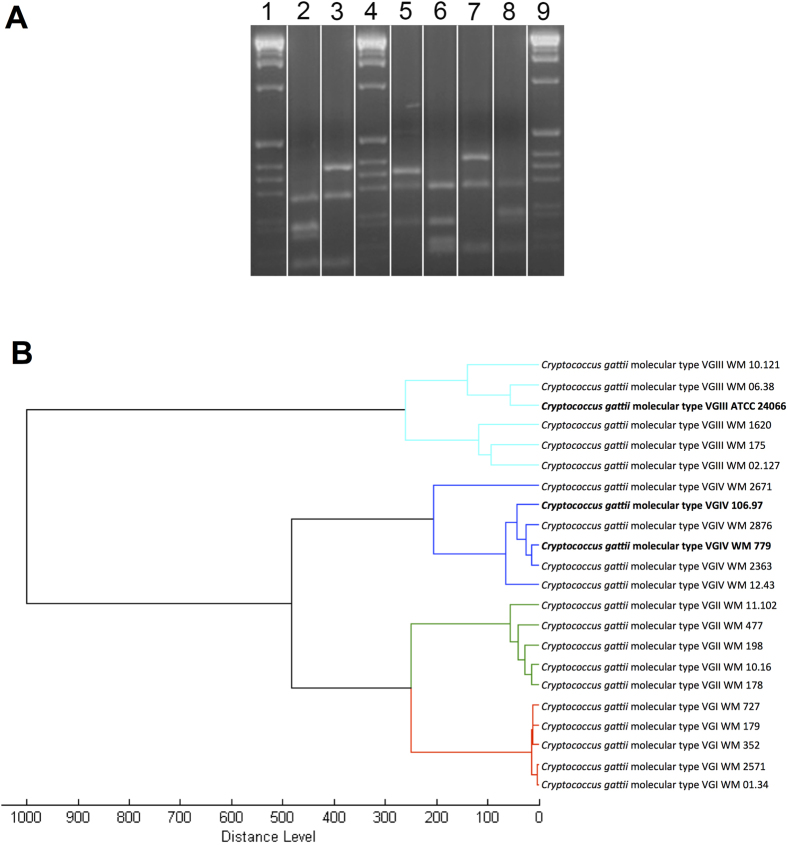
Molecular type and MALDI-TOF analyses of the *C. gattii* isolates. **A**. Molecular type analysis. Determination of the major molecular types via electrophoretic separation of *URA5* gene restriction patterns after double digestion with *Hha*I and *Sau*96I obtained from isolates 106.97 (VGIV (2)), ATCC 24066 (VGIII (3)), WM179 (VGI reference strain (5)), WM178 (VGII reference strain (6)), WM175 (VGIII reference strain (7)) and WM 779 (VGIV reference strain (8)), 1, 4, 9 = 1 Kb extension ladder (Invitrogen, USA). The gel showed in this panel is representative of different experiments performed under the same experimental conditions. Cropping lines are indicated in white and the unedited gel is available as Supplemental Figure 1. **B**. MSP dendrograms grouping mass spectra of *C. gattii* isolates according to their major molecular type. Isolates used in this analysis other than the three currently studied (bold) were obtained from the in-house MALDI_Biotyper BDAL MSP library at Westmead Hospital, Westmead Millennium Culture Collection at Sydney University – Sydney Medical School^32^.

Isolate WM 779 has been characterized before as belonging to the molecular type VGIV^30^, but the genotypic classification of the isolates 106.97 and ATCC 24066 of *C. gattii* were not known. That is why, the molecular types of those two isolates were determined via comparative *URA5*-RFLP analysis with the restriction enzymes *Hha*I and *Sau*96I with previously defined molecular types of the reference isolates of the major molecular types of *C. gattii* (Fig. 1A)^31^. Isolates ATCC 24066 and 106.97 were classified as molecular types VGIII and VGIV, respectively.

To verify the molecular type association obtained by *URA5*-RFLP analysis, Matrix-Assisted Laser Desorption Ionization-Time-of-Flight Mass Spectrometry (MALDI-TOF MS) was used, as it is a simple and reproducible method allowing the separation of the all major molecular types within the *C. neoformans/C. gattii* species complex^29^,^32^,^33^,^34^. The MALDI TOF profile of isolate WM 779, the global standard for the VGIV molecular type^10^, was recently described^32^,^33^. The MALDI-TOF MS profiles of isolates ATCC 24066 and 106.97 were generated in the current study. Identification of *C. gattii* isolates by MALDI-TOF generates consistency score values in the range of 2.3 to 3 for highly probable species identification, 2 to 2.29 for secure genus identification associated with probable species identification, 1.7 to 1.99 for probable genus identification and zero to 1.69 for not reliable identification^32^. In our study, all isolates were in the range of 2 to 2.29, indicating reliable identification of *C. gattii* (Table 1). MALDI-TOF spectra confirmed molecular types VGIV and VGIII for isolates 106.97 and ATCC 24066, respectively (Table 1). All studied *C. gattii* isolates grouped on the basis of MALDI-TOF MS spectra according to molecular types (Fig. 1B).

**Figure 2:**
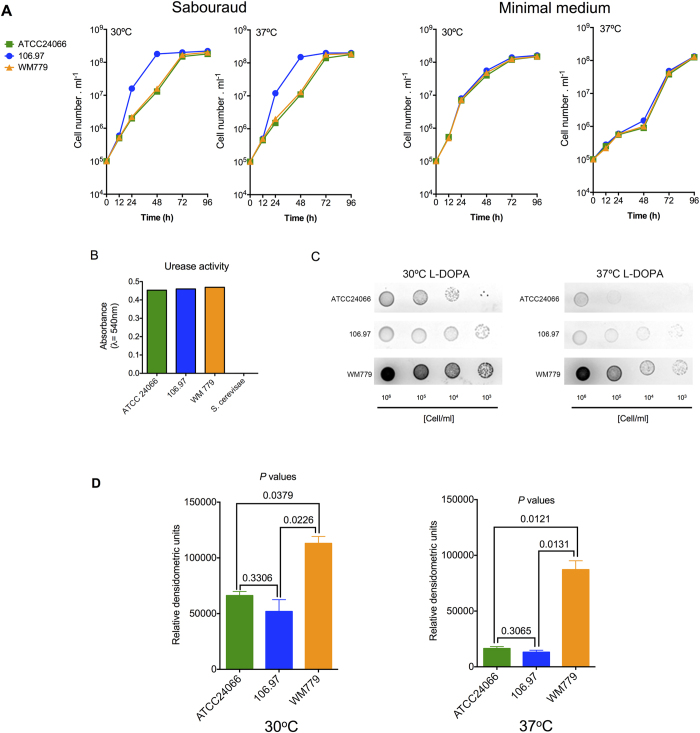
General phenotypic analysis of *C. gattii*. **A**. Growth curves of each isolate in Sabouraud broth or in minimal medium at both 30 ^o^C and 37 ^o^C. **B**. Urease activity from the three serotype C isolates and from *S. cerevisiae* control strain RSY113. **C.** Visual analysis of pigmentation after fungal growth in solid media supplemented with L-DOPA in different temperatures. **D**. Quantitative analysis of pigmentation demonstrated that isolate WM 779 was significantly more efficient in producing pigments than isolates 106.97 and ATCC 24066.

We also analyzed the ability of each isolate to replicate under different conditions and to produce key cryptococcal virulence factors. Growth curves of the three isolates at 30 °C and 37 ^o^C in Sabouraud broth revealed that cells of the isolate 106.97 had slightly faster growth kinetics, although all isolates reached the same cell number after 96 h of cultivation (Fig. 2A). In minimal medium, all isolates had similar growth curves (Fig. 2A). Urease activity was similar in all three isolates (Fig. 2B). Visual analysis of melanin production by the three *C. gattii* isolates revealed that after 120 h of cultivation in the presence of L-DOPA, the 106.97 and ATCC 24066 isolates had reduced rates of pigmentation at both 30 °C or 37 °C, in comparison with the WM 779 isolate (Fig. 2C). Quantification of pigmentation confirmed the visual analysis of melanin production (Fig. 2D).

### Capsular properties of *C. gattii* isolates

**Figure 3:**
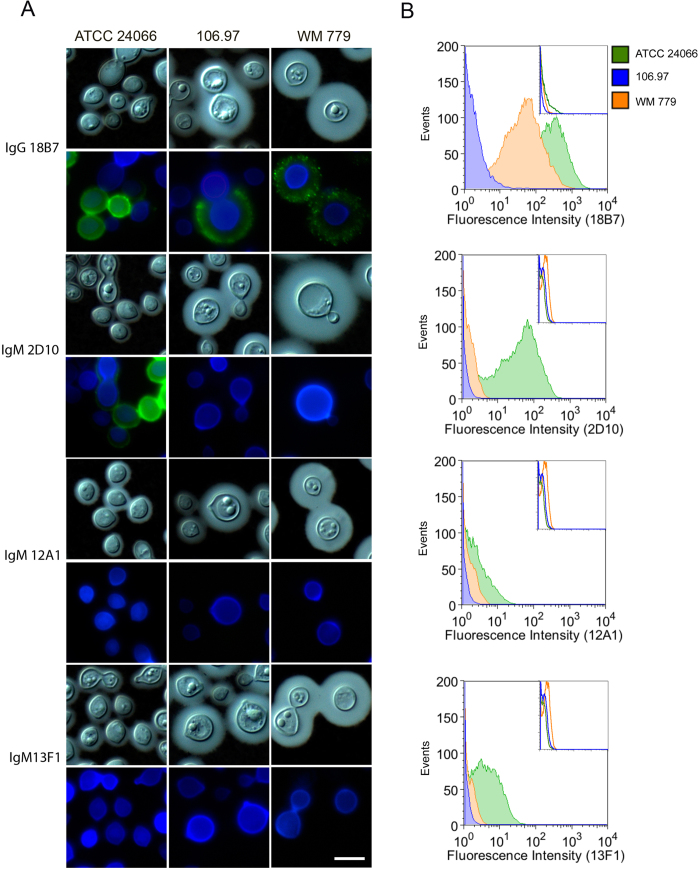
Reactivity of surface GXM with different mAbs. **A**. Microscopic analysis of fungal cells after sequential staining with GXM mAbs (green), calcofluor White (blue) and counter-staining with India ink. Each mAb used for GXM detection is indicated on the left. Scale bar: 5 μm. **B**. Flow cytometry analysis of the reactivity of GXM-binding mAbs with the serotype C *C. gattii* isolates. Insets denotes the fluorescence of fungal cells in systems where primary antibodies were not used.

Initially general aspects of morphology and serologic properties of the three *C. gattii* isolates were characterized (Fig. 3A). All three isolates produced capsule, but ATCC 24066 cells had much smaller capsular dimension. Immunofluorescence tests revealed that isolate 106.97 did not react with any of the antibodies raised against GXM. Assays using mAb 18B7 demonstrated reactivity only with isolates ATCC 24066 and WM 779 resulting annular and punctuate patters, respectively. MAb 2D10 showed annular reactivity with isolate ATCC 24066 and gave negative results for the WM 779 isolate. MAbs 12A1 and 13F1 produced negative serological reactions with all isolates. The results were confirmed in quantitative flow cytometry tests (Fig. 3B), revealing an unexpected diversity in the serological properties of the three *C. gattii* isolates analyzed here. This led us to investigate the carbohydrate compositions of polysaccharide extracts obtained from each isolate, as well as, to analyze the expression of capsule-related genes.

**Figure 4:**
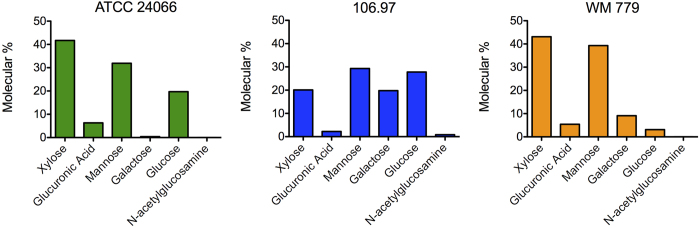
Glycosyl composition of polysaccharide extracts from the *C. gattii* isolates analyzed in this study. Monosaccharides were obtained after methanolysis of polysaccharide extracts for further GC-MS analysis, as detailed in the Methods section.

Capsular polysaccharides were extracted with DMSO and the resulting fractions had their glycosidic composition determined by GC-MS (Fig. 4). All isolates had similar xylose : glucuronic acid : mannose ratios, which was in accordance with previous studies determining the composition of capsular extracts from isolates belonging to VGIII and VGIV molecular types (serotype C)^35^. Other sugar units, however, varied in each isolate. Galactose, which was detected in trace amounts in capsular samples from isolate ATCC 24066, was abundant in fractions obtained from isolates 106.97 and WM 779. Glucose was highly concentrated in the samples obtained from isolates ATCC 24066 and 106.97, but not in the glycan fraction extracted from isolate WM 779. Finally, *N*-acetylglucosamine, which was absent in ATCC 24066 and WM 779 samples, was present at the trace level in fractions extracted from isolate 106.97.

**Figure 5:**
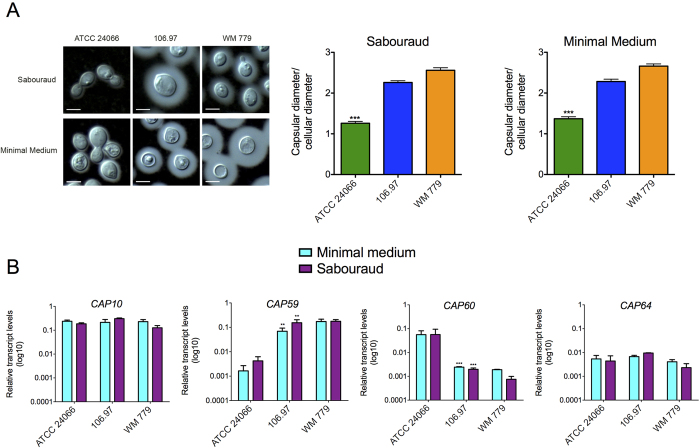
Capsular dimensions and expression of *CAP* genes in *C. gattii*. **A**. India ink counter-staining (left panels) and corresponding capsular dimensions (right panels) of *C. gattii* after growth in Sabouraud or minimal media. Capsular dimensions of the ATCC 24066 isolate were significantly smaller than those observed for 106.97 and WM 779 isolates (***p = <0.0005). Scale bar: 5 μm. **B**. Expression of *CAP* genes under the conditions used for determination of capsular dimensions. *CAP59* and *CAP60* had their expression significantly altered in the ATCC 24066 isolate (***p = <0.0001; **p = <0.005).

Results obtained from serological, morphological and glycosidic composition analyses clearly indicated a high diversity in capsular structures of the herein analyzed *C. gattii* isolates. To investigate the possibility of additional diversity at the molecular level, we analyzed the relationship between capsular dimensions and expression of *CAP* genes in the three isolates after 48 of cultivation in either Sabouraud broth or minimal medium. Cultivation of cryptococci in Sabouraud’s medium is known to repress capsule formation, while large capsules are usually formed by cryptococcal cells in minimal medium^36^. In comparison with the ATCC 24066 isolate, 106.97 and WM 779 cells showed larger capsular diameters after growth in both Sabouraud and minimal media (Fig. 5A). To further explore these differences, we used q-RT-PCR to determine the relative expression of the *CAP* family of capsule-related genes (*CAP59*, *CAP10*, *CAP60* and *CAP64*; Fig. 5B). *CAP64* and *CAP10* were similarly expressed in all isolates and conditions. On the other hand, expression of *CAP59* was reduced in isolate ATCC 24066, in both Sabouraud and minimal media. The same isolate showed enhanced expression of *CAP60* under similar conditions (Fig. 5B).

### The *C. gattii* isolates differ in chitooligomer detection and induction of host chitinase

**Figure 6:**
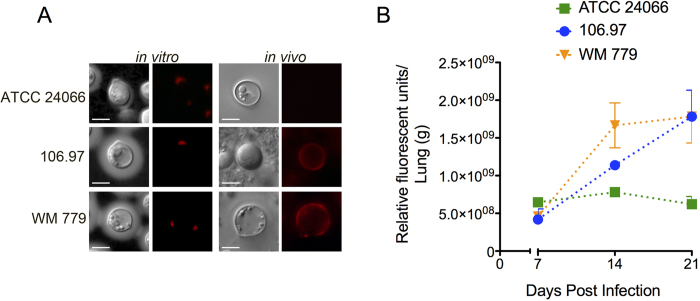
Analysis of *C. gattii* chitooligomers and host-derived chitinase activity. **A**. *In vitro* and *in vivo* detection of chitooligomers using TRITC-WGA. The typical polarized pattern of chitooligomer detection was observed *in vitro*. *In vivo* analysis revealed annular patterns of oligomer detection in isolates 106.97 and WM 779 and negative staining for the ATCC 24066 isolate. Scale bar, 5 μm. **B**. Pulmonary chitinase activity after infection with *C. gattii*. From day 14 to 21 post infection, chitinase activity was significantly higher when mice was infected with isolates 106.97 or WM 779, in comparison to the ATCC 24066 isolate (P < 0.05).

Chitooligomers are readily detectable components of the cell wall-capsule interface in both *C. neoformans* and *C. gattii* (molecular type VGII)^25^. These structures participate in the architecture^37^ and biological functions^38^ of capsular components. We therefore evaluated whether chitooligomer detection varied among the three isolates studied here. Microscopic analysis of *C. gattii* cells grown *in vitro* revealed that all three isolates had the staining profile that was previously observed in *C. gattii* serotype B and *C. neoformans* serotype A isolates^25^, consisting of polarized regions of the cell wall recognized by WGA (Fig. 6A). Considering that the profile of chitooligomer detection *in vivo* varies depending on the induction of pulmonary chitinases^37^, we also tested the distribution of GlcNAc oligomers in these three isolates after infection of mice. In this analysis, 106.97 and WM 779 cells obtained from the lungs of infected animals had an annular pattern of chitooligomer staining. However, similar experiments with the ATCC 24066 isolate gave negative results for chitooligomer staining. We also tested the levels of chitinase activity in the lungs of infected mice (Fig. 6B). *C. gattii* 106.97 and WM 779 (annular detection of chitooligomers) induced chitinase activity in a time-dependent fashion. In contrast, *C. gattii* ATCC 24066 isolate (no chitooligomer detection) induced chitinase activity at the lowest levels.

### *C. gattii* isolates of the VGIII and VGIV molecular types have different pathogenic potential

**Figure 7:**
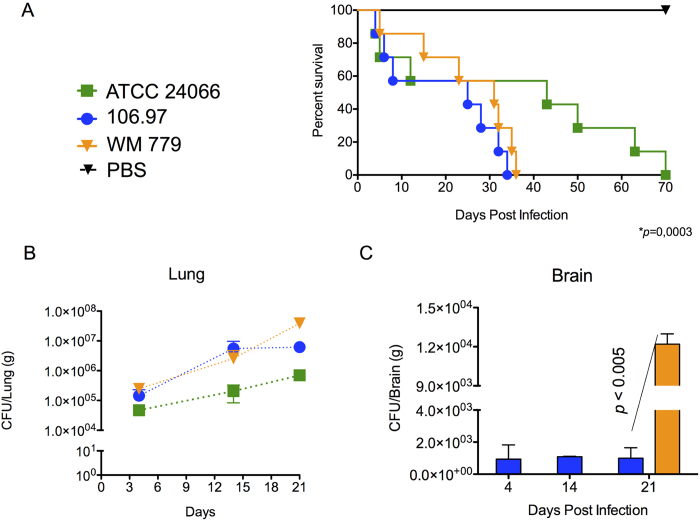
Mortality curves (**A**) and fungal burden (**B,C**) after infection of mice with serotype C isolates of *C. gattii*. **A.** Mice infected with the ATCC 24066 isolate lived longer than those infected with isolates 106.97 and WM 779 (*P* = 0.0003). **B.** Pulmonary burden after infection with isolate ATCC 24066 was always smaller (P<0.05) in comparison with systems where animals were inoculated with isolates 106.97 or WM 779. **C.** Brain colonization after infection of mice with isolates 106.96 and WM 779. Isolate WM 779 took longer to infect the brain of mice, but fungal burden 21 days post-infection was significantly higher for this isolate. Negative results were obtained when fungal colonization was analyzed after infection with isolate ATCC 24066. Isolate 106.97, blue bars; isolate WM 779, orange bars.

At this point, our results indicated that the *C. gattii* isolates studied herein differed in key determinants of the pathogenic potential observed within *C. gattii*. In summary, isolate ATCC 24066 differed from WM 779 and 106.97 cells in molecular type, capsule dimensions, expression of *CAP* genes, chitooligomer distribution, and induction of host chitinase, but additional diversity among the three isolates was also detected. For instance, isolate WM 779 was more efficient than the others in producing pigments, as already reported^20^, and all three isolates had distinct patterns of reactivity with antibodies to GXM. To evaluate whether this great diversity was translated into differential pathogenicity, we infected mice with the three isolates separately and compared host survival and fungal burden. Mortality curves (Fig. 7A) revealed that isolate ATCC 24066 was less efficient than the others in killing mice (*P* = 0.0003). Isolates WM 779 and 106.97 were similar in their ability to cause lethality and, in fact, they produced higher pulmonary fungal burden (Fig. 7B). Isolate ATCC 24066 was unable to reach the brain (data not shown), in contrast with 106.97 and WM 779 isolates (Fig. 7C). The later was only detected in the brain 21 days after infection, which contrasted with the cerebral detection of isolate 106.97 in days 4, 14 and 21 post-infection. However, after three weeks of infection, isolate WM 779 was approximately 100-fold more abundant in the brains of infected mice than 106.97.

## Discussion

Pathogenic diversity within microbial species has been known for decades^39^, but the molecular determinants regulating this phenomenon are multiple and, in most species, poorly known. In the *C. neoformans/C. gattii* complex, pathogenic diversity is important for the outcome of human disease, which has been correlated with cryptococcal-phagocyte interactions and laccase-dependent melanin pathways *in vitro*^40^. A recent study using an invertebrate model of infection revealed a formidable pathogenic diversity in 40 isolates of *C. gattii*, although the molecular determinants associated with this high variability remained undetermined^20^. The results described in the present study indicated remarkable differences in the pathogenicity of three isolates belonging to the same serotype (C), but to different molecular types (VGIII and VGIV) of *C. gattii*. The pathogenic determinants linked to these differences were mainly related to the surface architecture of *C. gattii* and included melanin formation, capsular structure and chitooligomer distribution.

One key virulence factor in *C. neoformans* and *C. gattii* is the ability to produce a polysaccharide capsule^41^. Mutants with defective capsule formation, in general, have avirulent phenotypes^21^,^42^,^43^. In our study, the less virulent isolate (ATCC 24066) manifested the lowest capsular dimensions, independently on the medium used for fungal growth. Accordingly, this phenotype was accompanied by decreased expression of *CAP59,* a gene that has been predicted to encode a protein required for GXM export^44^. Unexpectedly, *CAP60*, which has been described in seminal studies as one of the genes required for capsule formation^45^, was overexpressed in these cells. These results suggest that, during synthesis of capsular components and assembly of the capsule, the product of *CAP59* is required for the functions of *CAP60*. This hypothesis is further supported by the observation that *CAP60* and *CAP59*, despite their similarity in sequence and chromosome localization, cannot be functionally substituted by each other^45^.

Antibody reactivity with capsular components is essential for the classification of *Cryptococcus* species in different serotypes^46^. Our study demonstrated that serotype C isolates of *C. gattii* manifest an unexpected variability in their ability to react with GXM antibodies. These results support the notion that serotype classification of cryptococci does not reflect the phenotypic properties of different isolates. The less pathogenic isolate, ATCC 24066, was effectively recognized by two protective monoclonal antibodies, namely 18B7 and 2D10^47^,^48^. Isolate WM 779 was only recognized by mAb 18B7 and the 106.97 isolate had no reactivity with any of the antibodies tested here. This serologic variability imply that capsular structures and / or epitope distribution are diverse in at least two molecular types of *C. gattii* belonging to the same serotype (C) suggesting that the traditional classification of the *C. neoformans* species complex into four different serotypes may include still unknown capsular, serological determinants. In our study, carbohydrate analysis of capsular extracts revealed similar GXM composition, but the presence of secondary monosaccharides was different in each isolate. Therefore, the possibility that capsular components associate with other surface components resulting in modified serological reactivity cannot be ruled out. This hypothesis is supported by the recent demonstration that glycan association in the surface of *C. neoformans* resulted in hybrid polysaccharides with diverse immunological functions^38^.

The presence of fungal cells in brain tissue also varied in a murine model of cryptococcosis after infection with each isolate studied here. Only isolates 106.97 and WM 779, both VGIV isolates, disseminated to the brain, an observation that was linked to their ability to kill mice. A recent study by our group suggested that recognition of chitooligomers by macrophages is involved in dissemination of *C. neoformans* to the central nervous system^26^. In the present study, brain infection was in fact associated with increased chitooligomer distribution at the surface of fungal cells *in vivo* and elevated chitinase activity in the lung of infected mice. Pulmonary chitinase activity was linked with cryptococcal infection^49^ and increased exposure of surface fungal chitooligomers^37^. In addition, it has been recently demonstrated that chitinase-mediated chitin recognition induced pathologic responses to cryptococcal infection^50^. Therefore, we hypothesize that the increased chitooligomer exposure in isolates WM 779 and 106.97 results from their ability to induce pulmonary chitinase, in contrast to isolate ATCC 24066 (VGIII). This enhanced chitooligomer exposure could result in a more effective recognition of the fungus by macrophages, facilitating dissemination^26^. Finally, in our model, the highest brain fungal burden was observed 21 days after infection of mice with isolate WM 779, the most efficient in the production of melanin, as similarly reported among VGIV isolates^20^. Pigment synthesis has been linked with neurotropism, which is likely related to the high availability of diphenolic substrates used for pigment synthesis in the brain^51^. Melanin and fungal virulence are linked in many ways, since this pigment is required for protection of fungal cells against the antimicrobial arsenal produced by the host^52^,^53^. Importantly, the hypothetic importance of melanin formation for the virulence of cryptococci during human infection has been recently supported by the observation that laccase-dependent melanin pathways in different isolates are related to human clinical presentation and outcome^40^.

Variable virulence in *C. gattii* isolates has been suggested in a number of studies^3^,^16^,^20^,^54^. In a recent report with 40 globally selected strains including all molecular types, it has been demonstrated that virulence was not specifically associated with a particular molecular type of *C. gattii*, but rather with still undetermined attributes of each isolate analyzed^20^. Independently on the niche of isolation, some strains of the molecular types VGI, VGIII and VGIV were shown to be as virulent or even more virulent than the strains of the highly virulent subtype VGIIa, responsible for several fatal cases in the ongoing Vancouver Island outbreak^9^,^55^. The VGIV isolate WM 779, the one producing the highest fungal burden in the present study, was recently classified as a highly virulent strain^20^. In fact, we observed in our study that the two VGIV strains were more virulent than a VGIII isolate. The clinical relevance of this observation is still unknown, but human cases of meningitis caused by serotype C, VGIV, *C. gattii* have been reported in Mexico^56^ and India^57^. The molecular type VGIV also predominates in AIDS patients in Sub-Saharan Africa^58^.

Our work supports the notion that *C. gattii* isolates belonging to molecular types VGIII and VGIV are highly diverse in pathogenicity and phenotype. Although the molecular regulators of this potential diversity are still obscure, we propose that differences in melanin production, expression of *CAP* genes, capsular structure and surface architecture are involved in pathogenic diversity. These results reinforce the notion that even the serotype C VGIII and VGIV *C. gattii* isolates manifest hypervirulent phenotypes and open new venues for the investigation on how *C. gattii* cells cause damage to the host.

## Methods

Methods described in this section were carried out in accordance with the approved guidelines available in http://www.nature.com/srep/policies/index.html.

### Microorganisms and phenotypic characterization

The three *C. gattii* serotype C isolates used in this study included 106.97 (clinical isolate^35^), ATCC 24066 (clinical, reference isolate; American Type Culture Collection) and WM 779 (animal isolate, Westmead Millennium Culture Collection, The University of Sydney at Westmead Millennium Institute, Australia). Yeast cells were cultivated for 48 h at 30 °C with shaking in Sabouraud dextrose liquid medium (2% glucose, 1% peptone) and in a chemically defined minimal medium (pH 5.5) containing glucose (15 mM), MgSO_4_ (10 mM), KH_2_PO_4_ (29.4 mM), glycine (13 mM), and thiamine-HCl (3 μM). Growth rates were determined by counting the cell number in a Neubauer chamber in 24 h intervals. Yeast cells were harvested by centrifugation and washed in phosphate-buffered saline (PBS). Molecular typing was performed as described previously^31^. Analysis of the three isolates by MALDI-TOF MS was performed as recently described^32^,^33^. Three combined mass spectra (MSP) per isolate were generated on the MALDI-TOF Biotyper (BRUKER, Germany) using the MALDI Biotyper Automation Control software version 2.0.43.8 (BRUKER, Germany). For all morphological analyses, the cells were fixed with paraformaldehyde 4% for 30 min at room temperature and washed again in PBS, for further counter staining with India ink. Capsule to cell body diameter ratios (n = 100 cells) were determined using the Image J software (http://imagej.nih.gov). For determination of urease activity, the cells were grown for 24 h in liquid Sabouraud medium, washed three times with PBS and tested as described before^59^. *Saccharomyces cerevisae* (strain RSY113) was used as a negative control in these assays. Melanin production was determined visually after the cells were spotted on solid minimal medium supplemented with 1 mM l-dopa and cultivated for 120 h either at 30 ^o^C or 37 ^o^C^60^,^61^. Quantification of pigment formation was determined densitometrically after digitalization of the images generated for visual analysis.

### Analysis of expression of *CAP* genes

Each isolate was cultivated in liquid Sabouraud and in minimal media for 48 h as described above. Cells were collected by centrifugation for RNA extraction using the Trizol reagent (Invitrogen) according to the manufacturer’s protocol. Briefly, after DNAse treatment, RNA preparations were submitted to cDNA synthesis using M-MLV reverse transcriptase enzyme (Promega). Real-time PCR reactions were performed in an Applied Biosystems StepOnePlus Real-Time PCR System. Thermal cycling conditions consisted of an annealing step at 95 °C for 15 s followed by an elongation step at 60 °C for 1 min along for 40 cycles. Platinum SYBR Green qPCR SuperMix-UDG with ROX (Invitrogen) was used with 4 pmol of each primer and 2 μl of the cDNA template in a final reaction volume of 20 μl. Each sample was analyzed in triplicate. Melting curve analyses were performed at the end of each reaction. Results were normalized by using yeast ß-actin gene amplification. Relative expression was determined by the 2^−ΔCT^ method described by Livak & Schmittgen, 2001^62^.

### Determination of glycosidic composition in cellular glycans

Cellular polysaccharides were extracted with dimethyl sulfoxide (DMSO), using protocols previously established for efficient removal of GXM from *C. neoformans* cells^63^. Glycosidic composition was performed by combined gas chromatography/mass spectrometry (GC/MS) of the per-*O*-trimethylsilyl (TMS) derivatives of the monosaccharide methyl glycosides produced from the sample by acidic methanolysis according to previously described methods^64^.

### Fluorescence-based analysis of the cell surface

General properties of the cell surface of *C. gattii* were analyzed by fluorescence microscopy. Yeast cells (10^7^) were fixed in 4% paraformaldehyde (1 h, room temperature), blocked with 1% (w/v) BSA in PBS (1 h, 37 °C) and subsequently incubated with four different monoclonal antibodies (mAbs) raised to GXM, which were kindly donated by Dr. Arturo Casadevall (Baltimore, USA). The mAbs used in this study were: IgG1 18B7^47^ and IgMs 2D10, 13F1 and 12A1^44^,^47^. All mAbs were incubated with *C. gattii* at a final concentration of 10 μg/ml (1 h, 37 °C). The cells were subsequently incubated with Alexa Fluor™ 488-conjugated secondary antibodies (Invitrogen; 10 μg/ml, 1 h, 37 °C) and washed with PBS. Stained cells were analyzed by flow cytometry using a FACScan apparatus. Data was analyzed using the FlowJo7 software (FlowJo© Tree Star, Inc.). Alternatively, stained cells were probed with Calcofluor White at 5 μg/ml for 30 min (37 °C). The cells were finally counter-stained with India ink for analysis of capsular dimensions. After further washing with PBS, the cells were observed under a fluorescence optical microscope Axioplan 2 (Zeiss, Germany). Images were processed using the analysis software (Zen 2.0) and Adobe Photoshop CS5 was used for preparing figure panels.

### *In vivo* assays

BALB/c (female) mice (n = 7) approximately 4–6 weeks old were used for determination of mortality curves, fungal burden, pulmonary microscopic examination and chitinase activity after infection with *C. gattii*. The animals were anesthetized with ketamine (10 mg/kg) and xylazine (4 mg/kg) and inoculated intranasally with a fungal suspension of 10^5^ yeast cells in a final volume of 50 μl PBS. Groups were observed twice a day for 70 days for determination of mortality rates. Alternatively, groups of 12 mice were infected as described above with each of the isolates studied here. After 4, 14 and 21 days post infection, animals (n = 4) infected with each isolate were sacrificed and the lungs and the brains were excised and homogenized. These macerates were plated on Sabouraud medium for colony forming units (CFU) determination as previously described^65^. Lung macerates were also used for determination of chitinase activity. The homogenized pulmonary tissues were clarified by centrifugation for removal of cells and debris. Pelleted cells were stained with the wheat germ lectin (WGA) conjugated to tetramethylrhodamine (TRITC) at 5μg/ml for 30min (37°C). Supernatants were assessed for chitinase activity using the Chitinase Assay Kit Fluorimetric (CS1030 - Sigma Aldrich) according to the manufacturer instructions. Briefly, 5 μl of each sample were diluted to 100 μl with the commercial working substrate, for further incubation for 1 h at 37 °C. Reactions were read fluorimetrically as indicated by the manufacturer. The use of animals in this study was carried out with the approval of the Ethics Committees for animal use at the Federal Universities of Rio Grande do Sul and Rio de Janeiro (project number 093/14). The animals were kept in groups of up to 4 individuals, in cages with food and water *ad libitum*. Animals were maintained following the rules of the National Council of Animal Experimentation Control (CONCEA) and the Brazilian College of Animal Experimentation (COBEA).

### Statistical Tests

Student’s *t* test for comparison of sample data and the Kaplan-Meier survival statistics were carried out using the GraphPad Prism 6.0 software (GraphPad Software).

## Additional Information

**How to cite this article**: Rodrigues, J. *et al.* Pathogenic diversity amongst serotype C VGIII and VGIV *Cryptococcus gattii* isolates. *Sci. Rep.* **5**, 11717; doi: 10.1038/srep11717 (2015).

## Supplementary Material

Supplemental Figure 1

## Figures and Tables

**Table 1:** MALDI-TOF MS identification (ID) of the *C. gattii* isolates analyzed in this study.

Strain	Organism (best match)	Molecular type	Score value^a^
106.97	*C. gattii*	VGIV	2.276
ATCC 24066	*C. gattii*	VGIII	2.003
WM 779	*C. gattii*	VGIV	2.128

^a^ID scores are classified in 2.300 to 3.000 for highly probable species identification; 2.000 to 2.299 for secure genus identification with probable species identification; 1.700 to 1.999 for probable genus identification; and 0.000 to 1.699 for not reliable identification of *C. gattii*^32^.
