# Supplementary material for: Pathogenic diversity amongst serotype C VGIII and VGIV Cryptococcus gattii isolates
Source: Sci Rep. 2015 Jul 8;5:11717. doi: 10.1038/srep11717 (PMC4495446; doi:10.1038/srep11717)
Supplement: Supplemental Figure 1 [file srep11717-s1.pdf]

Pathogenic diversity amongst serotype C VGIII

and VGIV *Cryptococcus gattii* isolates

Jéssica Rodrigues, Fernanda L. Fonseca, Rafael O. Schneider, Rodrigo M. da C. Godinho, Carolina Firacative, Krystyna Maszewska, Wieland Meyer, Augusto Schrank, Charley Staats, Livia Kmetzsch, Marilene H. Vainstein, Marcio L. Rodrigues

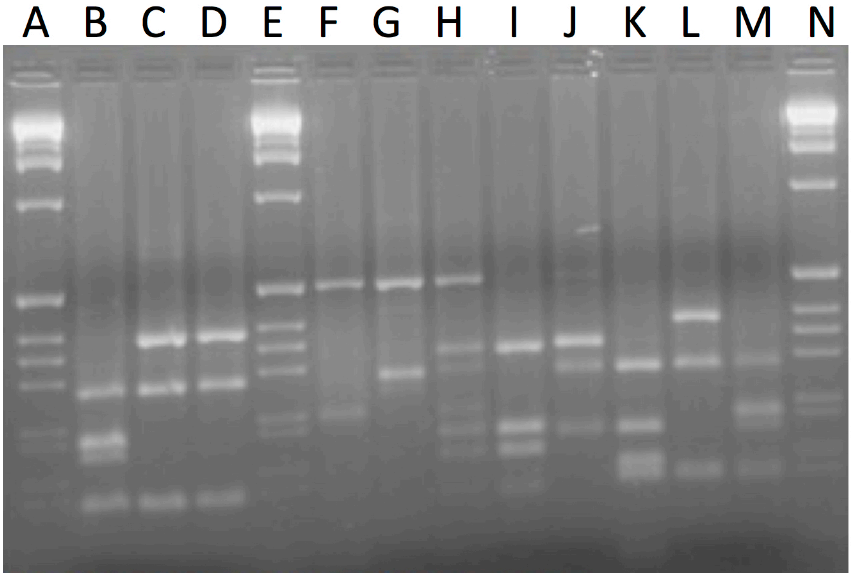

Supplemental Figure 1. Determination of the major molecular types via electrophoretic separation of *URA5* gene restriction patterns after double digestion with *Hha*I and *Sau*96I obtained from strains 106.97 (VGIV; B), HEC 40143 (VGIII; C), ATCC 24066 (VGIII; D), WM 148 (VNI; F), WM 626 (VNII; G), WM 628 (VNIII; H), WM 629 (VNIV; I), WM 179 (VGI; J), WM 178 (VGII; K), WM 161 (VGIII; L), WM 779 (VGIV; M). Lanes A, E and N represent 1 kb molecular markers.
